# Hypothermic oxygenated perfusion attenuates DCD liver ischemia–reperfusion injury by activating the JAK2/STAT3/HAX1 pathway to regulate endoplasmic reticulum stress

**DOI:** 10.1186/s11658-023-00466-5

**Published:** 2023-07-12

**Authors:** Pengpeng Yue, Xiaoyan Lv, Jian You, Yongkang Zou, Jun luo, Zhongshan Lu, Hankun Cao, Zhongzhong Liu, Xiaoli Fan, Qifa Ye

**Affiliations:** 1 Zhongnan Hospital of Wuhan University, Institute of Hepatobiliary Diseases of Wuhan University, Transplant Center of Wuhan University, National Quality Control Center for Donated Organ Procurement, Hubei Key Laboratory of Medical Technology on Transplantation, Hubei Clinical Research Center for Natural Polymer Biological Liver, Hubei Engineering Center of Natural Polymer-based Medical Materials, 430071 Wuhan, China; 2 Department of Hematology, Zhongnan Hospital of Wuhan University, Wuhan, 430071 China; 3 The Third Xiangya Hospital of Central South University, Research Center of National Health Ministry On Transplantation Medicine Engineering and Technology, Changsha, 410013 China

**Keywords:** Liver transplantation, DCD, HOPE, IRI, JAK2/STAT3, HAX1, Endoplasmic reticulum stress

## Abstract

**Background:**

Hepatic ischemia–reperfusion injury (IRI) in donation after cardiac death (DCD) donors is a major determinant of transplantation success. Endoplasmic reticulum (ER) stress plays a key role in hepatic IRI, with potential involvement of the Janus kinase 2/signal transducer and activator of transcription 3 (JAK2/STAT3) pathway and the antiapoptotic protein hematopoietic-lineage substrate-1-associated protein X-1 (HAX1). In this study, we aimed to investigate the effects of hypothermic oxygenated perfusion (HOPE), an organ preservation modality, on ER stress and apoptosis during hepatic IRI in a DCD rat model.

**Methods:**

To investigate whether HOPE could improve IRI in DCD livers, levels of different related proteins were examined by western blotting and quantitative real-time polymerase chain reaction. Further expression analyses, immunohistochemical analyses, immunofluorescence staining, terminal deoxynucleotidyl transferase-mediated dUTP nick end labeling (TUNEL) staining, and transmission electron microscopy were conducted to analyze the effects of HOPE on ER stress and apoptosis. To clarify the role of the JAK2/STAT3 pathway and HAX1 in this process, AG490 inhibitor, JAX1 plasmid transfection, co-immunoprecipitation (CO-IP), and flow cytometry analyses were conducted.

**Results:**

HOPE reduced liver injury and inflammation while alleviating ER stress and apoptosis in the DCD rat model. Mechanistically, HOPE inhibited unfolded protein responses by activating the JAK2/STAT3 pathway, thus reducing ER stress and apoptosis. Moreover, the activated JAK2/STAT3 pathway upregulated HAX1, promoting the interaction between HAX1 and SERCA2b to maintain ER calcium homeostasis. Upregulated HAX1 also modulated ER stress and apoptosis by inhibiting the inositol-requiring enzyme 1 (IRE1) pathway.

**Conclusions:**

JAK2/STAT3-mediated upregulation of HAX1 during HOPE alleviates hepatic ER stress and apoptosis, indicating the JAK2/STAT3/HAX1 pathway as a potential target for IRI management during DCD liver transplantation.

**Graphical Abstract:**

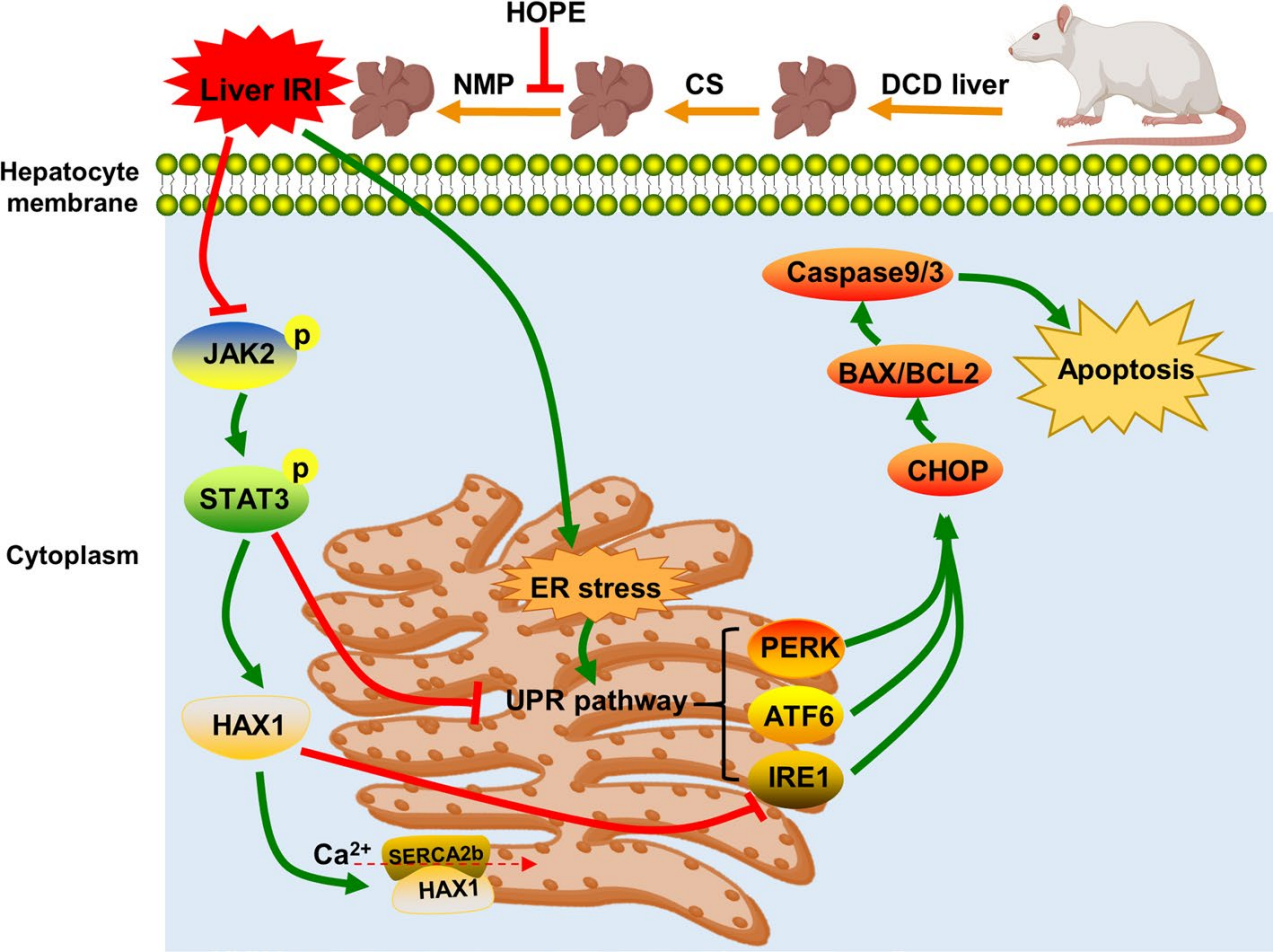

**Supplementary Information:**

The online version contains supplementary material available at 10.1186/s11658-023-00466-5.

## Background

Liver transplantation (LT) is the only effective treatment for end-stage liver diseases. However, there is a worldwide shortage of donor livers, resulting in a high mortality rate among patients awaiting transplantation [1]. The use of marginal donor livers included in the extended criteria donor (ECD) can effectively enlarge the donor liver pool [2, 3]. Among these, donation after cardiac death (DCD) donors can increase the number of donor livers by 20% [4]. However, early allograft dysfunction (EAD) and primary nonfunction (PNF) typically occur after DCD liver implantation, impairing long-term survival of the graft [5, 6]. Hepatic ischemia–reperfusion injury (IRI) is one of the primary reasons for poor prognosis in patients undergoing DCD liver transplantation [7, 8]. IRI is an inevitable injury to liver tissue after ischemia, hypoxia, and reperfusion during liver transplantation [1]. The DCD liver experiences a prolonged period of warm ischemia, causing severe IRI [3].

Among the current methods of organ preservation, dynamic mechanical perfusion (MP) has greater potential to reduce IRI than does cold storage (CS) [1]. Hypothermic oxygenated perfusion (HOPE) is a new method of dynamic organ preservation that aims to reduce the incidence and severity of IRI after organ transplantation [9]. Preclinical studies have shown that HOPE alleviates mitochondrial damage, oxidative stress damage, inflammation, and immune response [10–14]. Clinical trials demonstrated that HOPE can be used as a platform to evaluate the quality of donor livers, prevent tumor recurrence, reduce arterial and biliary complications, and improve graft survival and recipient prognosis [7, 15–17]. Compared with CS, HOPE is a safe and promising method for organ preservation, promoting graft survival.

Endoplasmic reticulum (ER) stress plays an important role in hepatic IRI [18, 19]. Particularly, hepatic warm ischemia and reperfusion cause Ca^2+^ overload, ATP deficiency, and oxidative stress, activating the ER stress response in hepatocytes [20]. A Ca^2+^ imbalance in the ER is considered to be one of the key events in cell death induced by ER stress, and Ca^2+^ plays a crucial role in ER stress [21]. Sarco/endoplasmic reticulum calcium ATPase (SERCA) is the key site for regulating Ca^2+^ balance in the ER. It transports Ca^2+^ from the cytoplasm to the ER lumen and maintains calcium homeostasis [22, 23]. The SERCA family has three subtypes: SERCA1, SERCA2 and SERCA3. SERCA1a and SERCA1b are mainly expressed in skeletal muscles; SERCA2a is expressed in the myocardium; SERCA2b is a subtype commonly expressed in almost all tissues, regulating Ca^2+^ homeostasis; and SERCA3 is expressed in vascular smooth muscle [24, 25]. Impaired SERCA activity can induce ER stress [22, 23]. To restore ER homeostasis, cells trigger an adaptive response called the unfolded protein response (UPR) [26]. The UPR is initiated by the dissociation of three main transmembrane proteins: inositol-requiring enzyme 1 (IRE1), activating transcription factor 6 (ATF6), and RNA-like ER kinase (PERK) [27]. Under emergency conditions, the accumulated unfolded proteins competitively replace the immunoglobulin heavy chain-binding protein (BIP) in IRE1, ATF6, and PERK, resulting in their activation in an attempt to reestablish homeostasis, restore ER homeostasis to the physiological state, and keep the cells alive [28]. However, if the ER stress response persists or if the protein load in the ER greatly surpasses its folding capacity, the cell’s ability to restore homeostasis is impaired. Consequently, the stress signaling pathway is redirected towards apoptosis, resulting in cell death [29, 30].

After continuous injury caused by warm ischemia, cold ischemia, and reperfusion, ER stress aggravates in the DCD liver, leading to hepatocyte apoptosis [31]. Although HOPE can alleviate DCD liver injury, its effect and potential mechanism of action on ER stress remain unclear. In this study, we established a DCD rat model and HOPE system to investigate the effect of HOPE on ER stress in the process of liver IRI in such rats, clarify its mechanism, and identify a new intervention target for the treatment of IRI during DCD liver transplantation.

## Methods

### Animals

Adult male Sprague–Dawley (SD) rats (8–10 weeks old, weighing 280–320 g) were obtained from Wan Qian Jia Xing Bio-Technology Co., Ltd. (Hubei, China) and raised in a standard feeding environment at the Animal Experimental Center of Zhongnan Hospital of Wuhan University. This study was approved by the Experimental Animal Welfare Ethics Committee of the Zhongnan Hospital of Wuhan University. All animal experiments were conducted in accordance with the regulations of the Experimental Animal Management Ordinance (National Science and Technology Committee of China) and the Guide for the Care and Use of Laboratory Animals (National Institutes of Health, Bethesda, MD, USA).

### Experimental design and DCD model construction

Pentobarbital sodium salt (30 mg/kg) administered intraperitoneally was used to anesthetize the rats. The DCD model was established as previously described [32, 33]. Briefly, the rats were fixed on a sterile operating table for skin preparation, disinfection, and toweling. Under conditions of no portal vein clamping or prior heparinization, 30 min of cardiac arrest was induced by cutting the diaphragm. During this period, the rats were placed on a constant temperature heating plate, and the anal temperature was monitored and kept at 29 ± 1.45 ℃. After warm ischemia, the liver was flushed through the abdominal aorta with 50 mL of histidine–tryptophan–ketoglutarate (HTK) solution at 4 ℃ [34]. The portal vein, superior hepatic vena cava, and common bile duct were intubated, and the inferior hepatic vena cava and right adrenal vein were ligated. Finally, the livers were dissected and removed.

The experimental groups were further divided into three groups (*n* = 6 for each group). Cardiac arrest was not induced in the sham group, but all other processes were performed as above. In the CS group, the isolated liver was stored in HTK solution at 4 ℃ for 24 h after successful DCD modeling. In the HOPE group, the liver was stored in HTK solution at 4 ℃ for 23 h and then subjected to HOPE for 1 h. All groups were rewarmed at room temperature for 15 min after the above operation and then subjected to normothermic perfusion (NMP) for 1 h. The establishment of HOPE and NMP systems have been described in detail previously [32, 33]. Perfusion and liver tissue samples were collected at the end of NMP.

Depending on the experimental requirements, AG490 was used in different groups. AG490 (10 mg/kg) or vector [5% dimethyl sulfoxide (DMSO) and 95% phosphate-buffered saline (PBS)] was injected intraperitoneally 2 h before constructing the DCD model.

### Cell culture and hypoxia/reoxygenation (H/R) model

BRL-3A cells were cultured in high-glucose medium containing 1% antibiotics and 10% fetal bovine serum at 37 ℃ and 5% CO_2_. To construct the H/R model, the cell culture medium was replaced with serum-free low-glucose medium and cultured in 37 ℃, 1% O_2_, 5% CO_2_, and 94% N_2_ for 24 h. Thereafter, the medium was replaced with high-glucose medium containing serum and cultured for 6 h at 37 ℃ and 5% CO_2_. According to the experimental design, the cells were treated with AG490 (10 μM) or vector.

### Plasmid construction and in vitro transfection

The rat hematopoietic-lineage substrate-1-associated protein X-1 (HAX1) overexpression plasmid was constructed by Genomeditech Co., Ltd. (Shanghai, China). The 2 μg plasmid was transiently transfected into 70% confluent cells in a six-well plate using NEOFECT DNA transfection reagent (Neofect, Beijing, China). The cells were then cultured for 24 h and harvested.

### Biochemical analysis, SERCA activity, and caspase-3 activity detection

Alanine aminotransferase (ALT) and aspartate aminotransferase (AST) levels were determined using an automatic biochemical analyzer (Chemray 80, Shenzhen, China). Intracellular SERCA activity was measured using a Ca^2+^-ATPase assay kit (Jiancheng, Nanjing, China) and normalized to protein concentration. The caspase-3 activity assay kit (Jiancheng, Nanjing, China) was used to detect the activity of caspase-3 enzyme in cell and tissue lysates.

### HE and TUNEL staining

Liver tissue samples were fixed in 4% paraformaldehyde, embedded in paraffin, sectioned, and stained with hematoxylin and eosin (H&E). Images were observed and captured using a light microscope. Grading was performed by three pathologists according to the Suzuki criteria [35]. Terminal deoxynucleotidyl transferase-mediated dUTP nick end labeling (TUNEL) staining was used to detect hepatocyte apoptosis, following the instructions of the kit. A fluorescence microscope (Nikon Eclipse C1, Japan) was used to observe and capture the images.

### Transmission electron microscope (TEM)

Fresh liver tissue samples (1–2 mm^2^) were fixed using TEM fixative (Servicebio, Wuhan, China) and washed three times with 0.1 M phosphate buffer (pH 7.4). The tissue was then dehydrated using a gradient of 30–100% ethanol and fixed with acetone. Afterward, the tissue was embedded in resin, sliced into sections measuring 60–80 μm, and placed on 150-mesh copper grids. To prevent CO_2_ staining, the sections were stained with a 2% uranium acetate saturated alcohol solution and a 2.6% lead citrate solution, followed by drying with filter paper. Finally, the images were observed using a TEM (HT7800, Hitachi, Japan), and the results were analyzed.

### Immunohistochemistry and immunofluorescence staining

Immunohistochemistry (IHC): The paraffin sections were blocked with 3% hydrogen peroxide and 3% bovine serum albumin (BSA) and incubated overnight with anticleaved caspase-3 antibody (1:100, Cell Signaling Technology, 9661, Massachusetts, USA) at 4 °C. The sections were incubated with secondary antibodies, subjected to DAB staining, re-stained for the nucleus, dehydrated, and finally observed and photographed under a light microscope.

Immunofluorescence (IF): Immunofluorescence was performed as previously reported [32]. Paraffin sections or cell-climbing slices were incubated with anti-CHOP (1:100, Abclonal, A0221, Wuhan, China), anti-BIP (1:500, Proteintech, 11587-AP, Wuhan, China), anti-HAX1 (1:100, Abclonal, A5551, Wuhan, China), or anti-SERCA2b (1:500, Proteintech, 67248–1-Ig, Wuhan, China) antibodies. Secondary antibodies labeled with CoraLite 488 (1:1000, Proteintech, SA00013-1, Wuhan, China) or CoraLite 594 (1:500, Proteintech, SA00013-4, Wuhan, China) were then incubated. After DAPI staining, cells were observed and photographed under a fluorescence microscope.

### Quantitative real-time PCR

Total RNA was extracted using the TRIzol reagent (Biosharp, Hefei, China), and cDNA was synthesized using a Script cDNA reverse transcription kit (Servicebio, Wuhan, China). Finally, real-time polymerase chain reaction (real-time PCR) was performed with SYBR Green (Servicebio, Wuhan, China). The sequences of the primers for the target genes are listed in Additional file 1: Table S1.

### Western blotting

Proteins were extracted from the liver tissues and cells. Sodium dodecyl sulfate–polyacrylamide gel electrophoresis (SDS–PAGE) was used to separate the protein according to its relative molecular weight, which was then electro-transferred to a polyvinylidene fluoride (PVDF) membrane. After being blocked by 5% BSA for 2 h, the membrane was incubated with the primary antibody at 4 ℃ overnight. After labeling with a secondary antibody (1:5000, goat anti-rabbit or goat anti-mouse, Abclonal, Wuhan, China), the protein bands were stained with enhanced chemiluminescence (ECL) reagent (Biosharp, Hefei, China), and protein expression was quantified using ImageJ software. The primary antibodies used in this study were as follows: anti-BIP (1:5000, Proteintech, 11587-1-AP, Wuhan, China), anti-CHOP (1:1000, Abclonal, A0221, Wuhan, China), anti-ATF6 (1:2000, Proteintech, 24169-1-AP, Wuhan, China), anti-ATF4 (1:1000, Proteintech, 10835-1-AP, Wuhan, China), anti-IRE1 (1:1000, Abclonal, A17940, Wuhan, China), anti-p-IRE1 (1:1000, Zen Biosciences, 530878, Wuhan, China), anti-XBP-1s (1:500, Abclonal, A22546, Wuhan, China), anti-BAX (1:10,000, Proteintech, 50599-2-Ig, Wuhan, China), anti-BCL2 (1:5000, Proteintech, 60178–1-Ig, Wuhan, China), anti-PUMA (1:1000, Abclonal, A3752, Wuhan, China), anti-NOXA (1:1000, Abclonal, A9801, Wuhan, China), anti-GADD45A (1:500, Abclonal, A13487, Wuhan, China), anti-Caspase-9 (1:1000, Proteintech, 10380-1-AP, Wuhan, China), anti-Caspase-3 (1:1000, Proteintech, 19677-1-AP, Wuhan, China), anti-JAK2 (1:1000, Abclonal, A11497, Wuhan, China), anti-p-JAK2 (1:1000, Abclonal, AP0531, Wuhan, China), anti-STAT3 (1:1000, Cell Signaling Technology, 30835, Massachusetts, USA), anti-p-STAT3 (1:2000, Cell Signaling Technology, 9145, Massachusetts, USA), anti-HAX1 (1:1000, Abclonal, A5551, Wuhan, China), anti-SERCA2b (1:10,000, Proteintech, 67248-1-Ig, Wuhan, China), and anti-β-actin (1:50,000, Abclonal, AC026, Wuhan, China) antibodies.

### Co-immunoprecipitation (CO-IP) analysis

Liver tissue protein was extracted, and 10 μg of anti-HAX1 antibody (Abclonal, A5551, Wuhan, China) was added to the lysate containing 500 μg of protein and incubated overnight at 4 ℃ to form a binding immune complex. Thereafter, 25 μL (0.25 mg) of protein A/G magnetic beads (Thermo Fisher, Shanghai, China) were added and incubated for 1 h at room temperature. The obtained complex was washed, eluted, denatured according to the manufacturer’s instructions, and analyzed by western blotting.

### Flow cytometry

Flow cytometry was performed to measure apoptosis rate and mitochondrial membrane potential. Cells were subjected to trypsin digestion and stained with an Annexin V-FITC apoptosis detection kit (Beyotime, Shanghai, China) to assess apoptosis and a JC-1 assay kit (Beyotime, Shanghai, China) to evaluate mitochondrial membrane potential. Analysis was carried out on a flow cytometer (CytoFlex S, Beckman, USA).

### Statistical analysis

Statistical analysis was performed using GraphPad Prism 8.0 software (GraphPad Prism Software, La Jolla, CA, USA). All data are presented as mean ± standard deviation. Differences between groups were analyzed using *t*-test and one-way analysis of variance (ANOVA). *p* < 0.05 was considered statistically significant.

## Results

### HOPE reduces liver injury and inflammation in DCD rat models

**Fig. 1:**
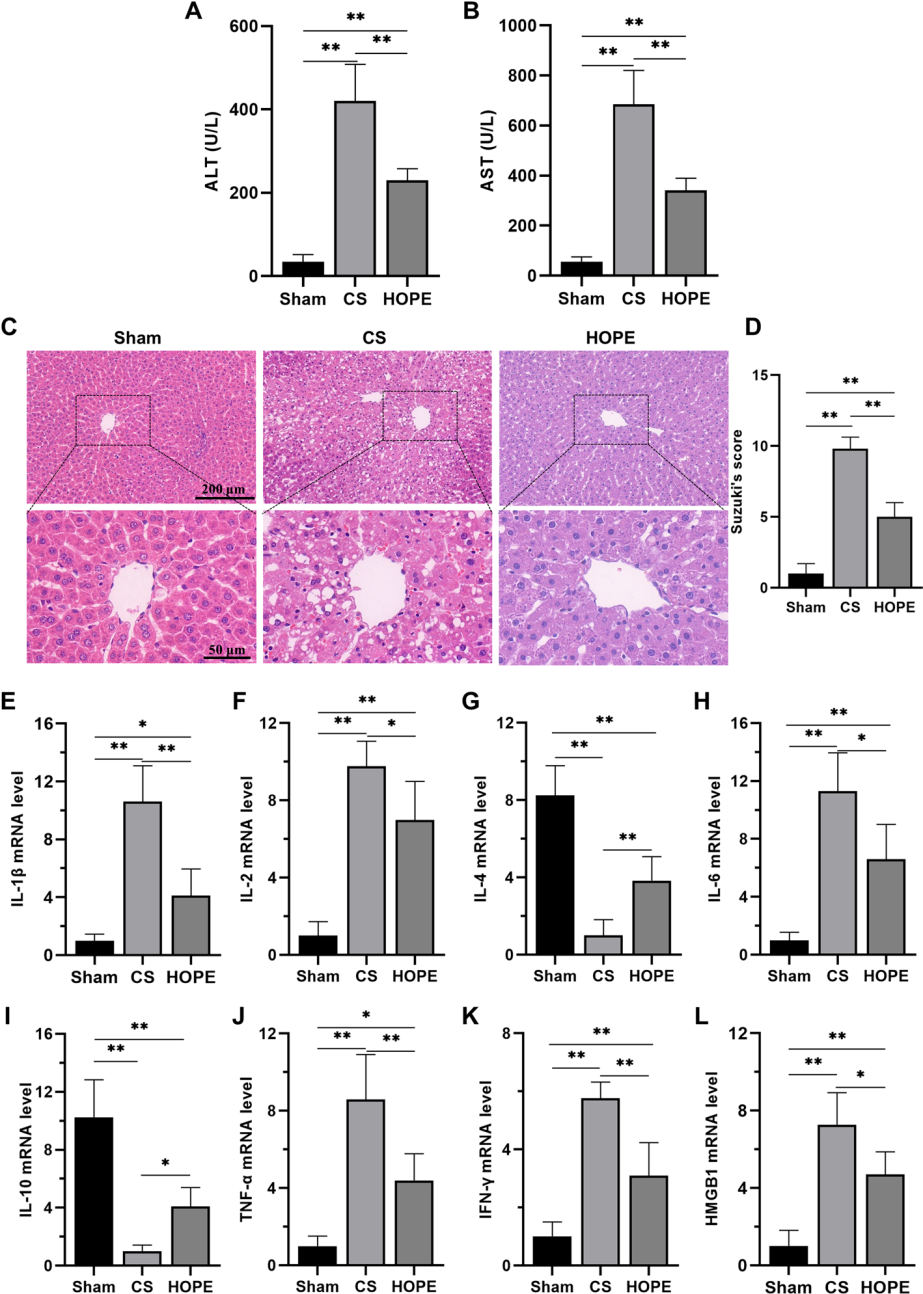
HOPE improves liver function and reduces inflammation in a rat model of DCD. **A**, **B** The levels of AST and ALT in the perfusate (*n* = 6 for each group). **C**, **D** H&E staining and histological injury score in rat liver tissue (*n* = 6 for each group). Scale = 200 μm (top panel) and 50 μm (bottom panel). **E**–**L** mRNA expression levels of IL-1β, IL-2, IL-4, IL-6, IL-10, TNF-α, IFN-γ, and HMGB1 (*n* = 3 for each group). ^*^p < 0.05 and ^**^ p < 0.01

To verify the effect of HOPE on improving IRI in DCD livers, we first measured the levels of ALT and AST in perfusates. The results revealed significantly elevated ALT and AST levels in the CS group compared with the sham group, which notably decreased following HOPE treatment (Fig. 1A, B). Second, we evaluated the pathological damage in the three groups. HE staining revealed that compared with those in the sham group, the liver tissues of the CS group exhibited obvious inflammatory cell infiltration, congestion, hepatocyte necrosis, and vacuolation, whereas these pathological injuries were significantly alleviated in the HOPE group liver tissues (Fig. 1C, D). These results suggest that HOPE significantly improves liver function and reduces liver tissue injury.

Inflammation plays an important role in IRI development. After warm ischemia, cold ischemia, and reperfusion, the DCD liver exhibited an obvious inflammatory reaction [36]. To demonstrate that HOPE can reduce the inflammatory reaction, we used qRT–PCR to detect the levels of proinflammatory factors (IL-1β, IL-2, IL-6, TNF-α, IFN-γ, and HMGB1) and antiinflammatory factors (IL-4 and IL-10). The results indicated that the levels of proinflammatory factors were significantly increased and those of antiinflammatory factors were significantly decreased in the CS group compared with in the sham group. The HOPE group showed a reversal of these changes after HOPE treatment (Fig. 1E–L). These results suggest that HOPE can significantly reduce the inflammatory response during IRI in the DCD livers.

### HOPE alleviates hepatic ER stress in DCD rat models

**Fig. 2:**
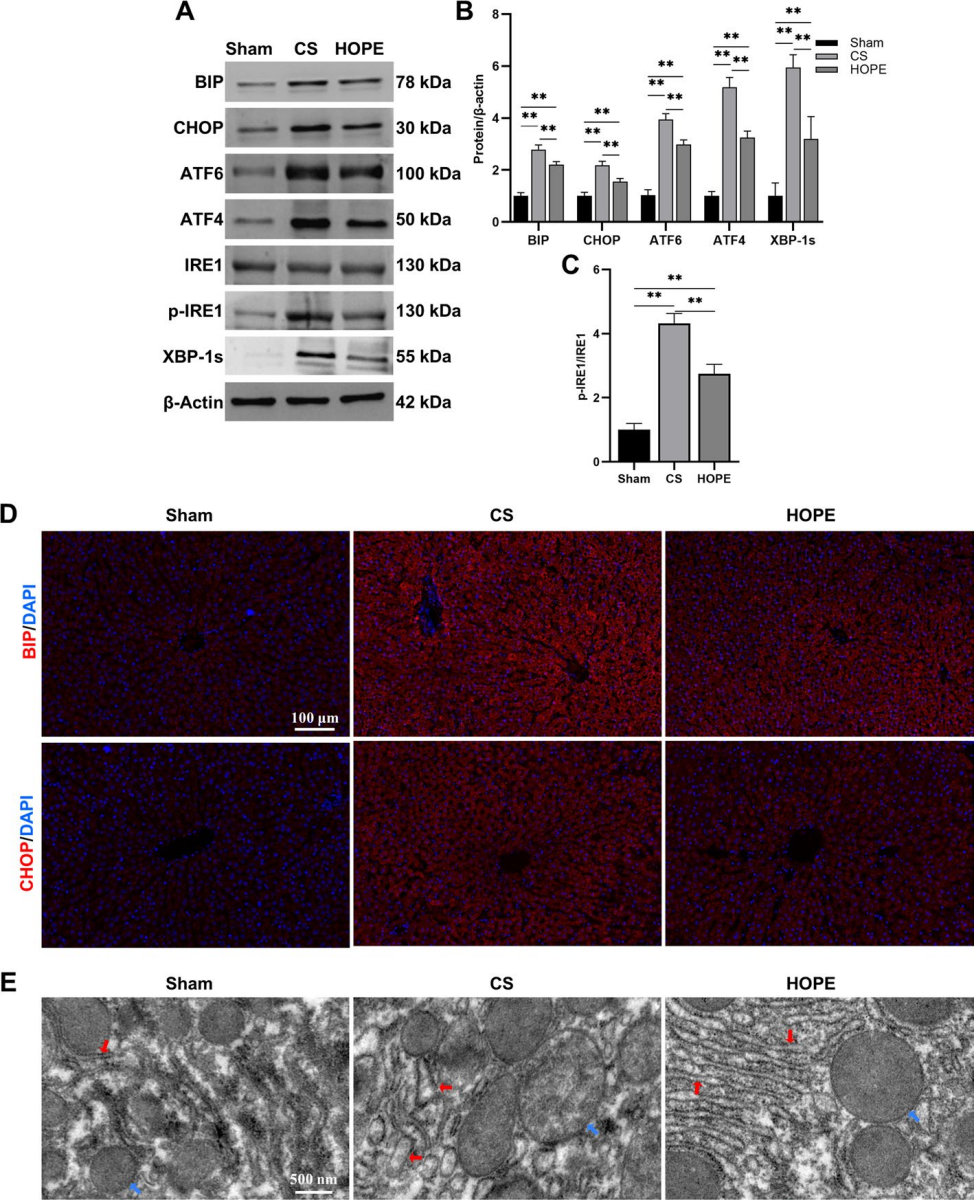
HOPE alleviates hepatic ER stress in a rat model of DCD. **A**–**C** Western blotting and statistical analyses of BIP, CHOP, ATF6, ATF4, IRE1, p-IRE1, XBP-1s, and β-actin proteins (*n* = 3 for each group). p-IRE1 indicates phospho-IRE1. **D** IF staining of BIP and CHOP proteins in rat liver tissues (*n* = 6 for each group). Scale = 100 μm. **E** The microstructure of hepatocytes as observed by TEM (*n* = 3 for each group). The red arrow indicates the ER, and the blue arrow indicates the mitochondria. Scale = 500 nm. ^*^p < 0.05 and ^**^p < 0.01

ER stress participates in the process of liver IRI and causes cell death [18, 19]. To investigate whether HOPE could reduce ER stress during IRI in the DCD liver, we assessed the activation of ER stress and the UPR signaling pathways. Western blot analysis showed that compared with those in the sham group, the expression of ER stress marker proteins CHOP and BIP, as well as those of the key signaling molecules ATF4 (a key molecule of the PERK pathway), ATF6, p-IRE1, and X-box binding protein l splicing (XBP-1s) (a key molecule of the IRE1 pathway) in the three UPR pathways were upregulated in the CS group, and this trend was significantly reduced by HOPE treatment (Fig. 2A–C). We also evaluated the mRNA levels of BIP and CHOP using qRT–PCR, and the trend was consistent with that of western blotting (Additional file 2: Fig. S1). Furthermore, immunofluorescence results demonstrated that cytoplasmic BIP and CHOP were significantly upregulated in the CS group and inhibited in the HOPE group (Fig. 2D). We also examined the ER structural changes using TEM. In the CS group, the ER cisternae showed significant expansion and structural damage compared with those in the sham group. However, such morphological alterations in the ER were minimal in the HOPE group (Fig. 2E). Additionally, HOPE treatment alleviated mitochondrial swelling that was observed in the CS group (Fig. 2E). These findings suggest that the DCD livers can induce ER stress and activate the UPR signaling pathways after CS, and that HOPE treatment can significantly inhibit this change.

### HOPE alleviates hepatic apoptosis in DCD rat models

**Fig. 3:**
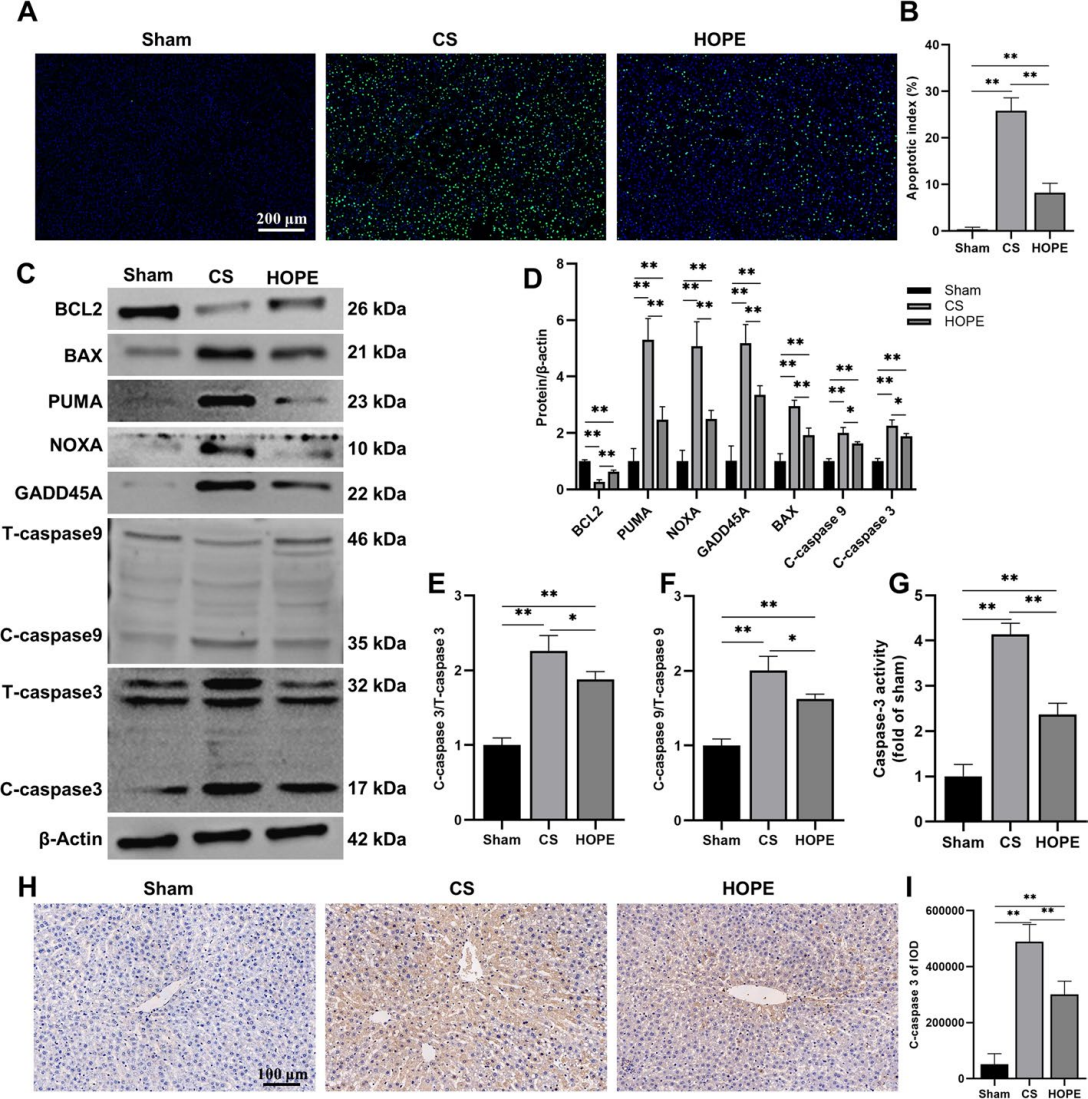
HOPE alleviates hepatic apoptosis in a rat model of DCD. **A**, **B** TUNEL staining and scoring of rat liver tissue (*n* = 6 for each group). Scale = 200 μm. **C**–**F** Western blotting and statistical analyses of BCL2, BAX, PUMA, NOXA, GADD45A, caspase-9, caspase-3, and β-actin proteins (*n* = 3 for each group). T-caspase indicates total caspase, C-caspase indicates cleaved caspase. **G** Caspase-3 activity detection (*n* = 6 for each group). **H**, **I** IHC staining and statistical analysis of C-caspase-3 protein in rat liver tissue (*n* = 6 for each group). Scale = 100 μm. ^*^p < 0.05 and ^**^p < 0.01

Under ER stress, cells reduce the load of unfolded proteins by expanding the ER, inducing the synthesis of key chaperone molecules, and reducing the synthesis of total proteins. When cells are damaged by irreparable ER stress, activation of the UPR signaling pathway directly induces apoptosis and eliminates injured cells [37–39]. PERK, ATF6, and IRE1 induce CHOP expression, which inhibits the expression of BCL2 and promotes the expression of apoptotic proteins [40]. Therefore, we evaluated the level of apoptosis in the three groups. First, TUNEL staining of liver tissue showed that the apoptosis level in the CS group livers was significantly upregulated compared with that of the sham group livers, whereas after HOPE treatment, the apoptosis rate significantly decreased (Fig. 3A, B). Next, we determined the expression of apoptosis-related proteins. CS significantly downregulated BCL2 and upregulated BAX, p53-upregulated modulator of apoptosis (PUMA), phorbol-12-myristate-13-acetate-induced protein 1 (PMAIP1/NOXA), growth arrest and DNA damage inducible alpha (GADD45A), cleaved-caspase-9, and cleaved-caspase-3. This trend was significantly reversed after HOPE treatment (Fig. 3C–F). The caspase-3 activity assay and IHC results showed that the activity and expression of caspase-3 was upregulated in the CS group and decreased in the HOPE group, which was consistent with the western blotting results (Fig. 3G–I). These results suggest that HOPE alleviates IRI-induced apoptosis in the DCD livers.

### HOPE attenuates ER stress and apoptosis by activating the JAK2/STAT3 pathway

**Fig. 4:**
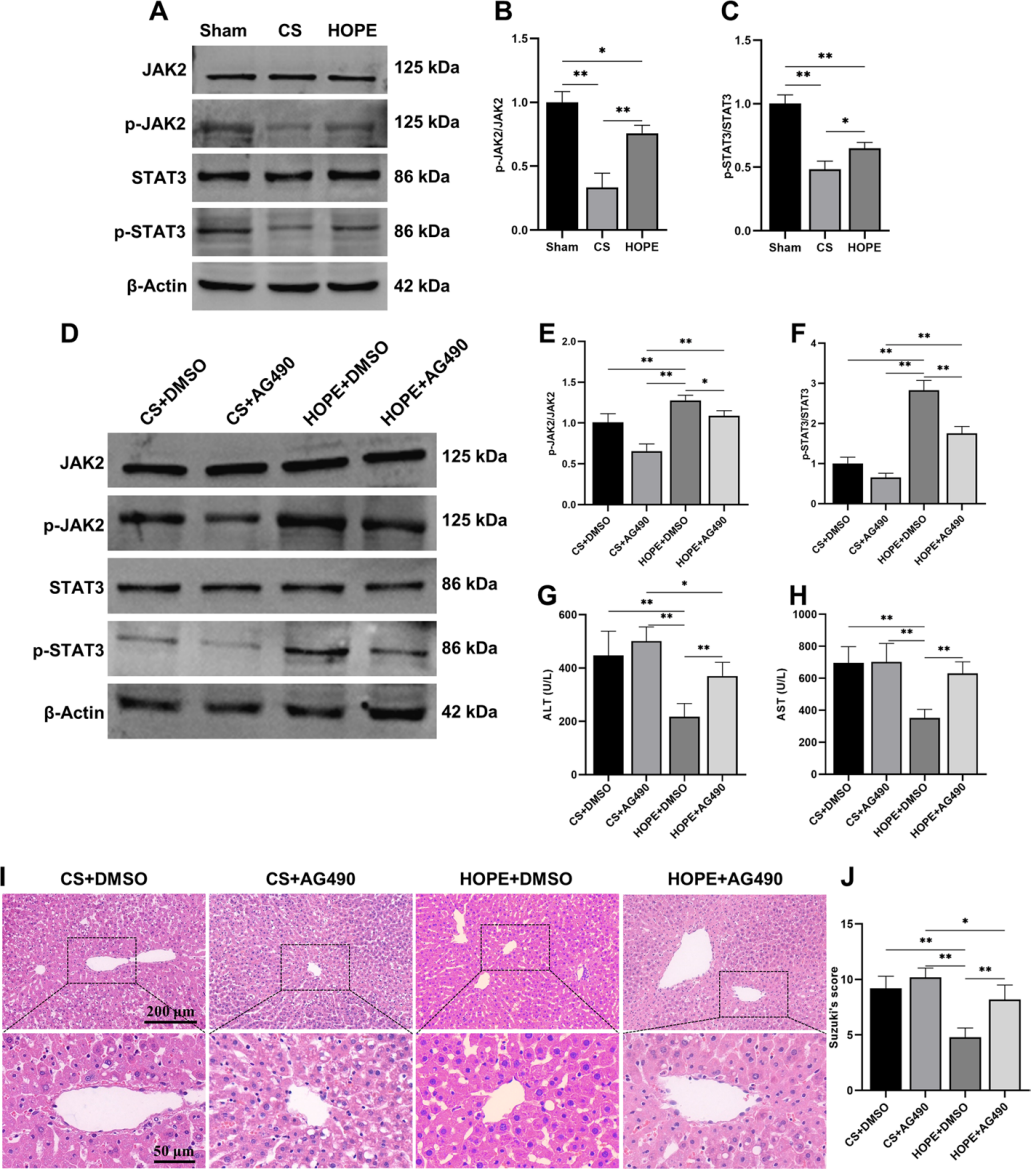
HOPE attenuates liver injury by activating JAK2/STAT3 pathway in a rat model of DCD. **A**–**F** Western blotting and statistical analyses of JAK2, p-JAK2, STAT3, p-STAT3, and β-actin proteins (*n* = 3 for each group). p-JAK2 indicates phospho-JAK2, p-STAT3 indicates phospho-STAT3. **G**, **H** The levels of AST and ALT in the perfusate (*n* = 6 for each group). **I**, **J** H&E staining and histological injury score in rat liver tissue (*n* = 6 for each group). Scale = 200 μm (top panel) and 50 μm (bottom panel). ^*^p < 0.05 and ^**^p < 0.01

The Janus kinase 2/signal transducer and activator of transcription 3 (JAK2/STAT3) pathway responds quickly to stress and play an important role in mediating liver IRI [37, 41–43]. Compared with the sham group, the phosphorylation of JAK2 and STAT3 in the CS group decreased significantly, and the phosphorylation level was significantly increased after HOPE treatment (Fig. 4A–C). To further determine the role of the JAK2/STAT3 pathway in DCD liver IRI, we pretreated rats with AG490, a specific inhibitor of the JAK2/STAT3 pathway [44]. The results showed that the HOPE-induced phosphorylation of JAK2 and STAT3 was significantly inhibited by AG490 (Fig. 4D–F). In addition, the decreased levels of ALT and AST in HOPE-treated rats were reversed by AG490 (Fig. 4G, H). HE staining showed that AG490 aggravated inflammatory cell infiltration, congestion, hepatocyte necrosis, and vacuolation (Fig. 4I, J). These results suggest that HOPE attenuates liver injury in a DCD rat model by activating the JAK2/STAT3 pathway.

**Fig. 5:**
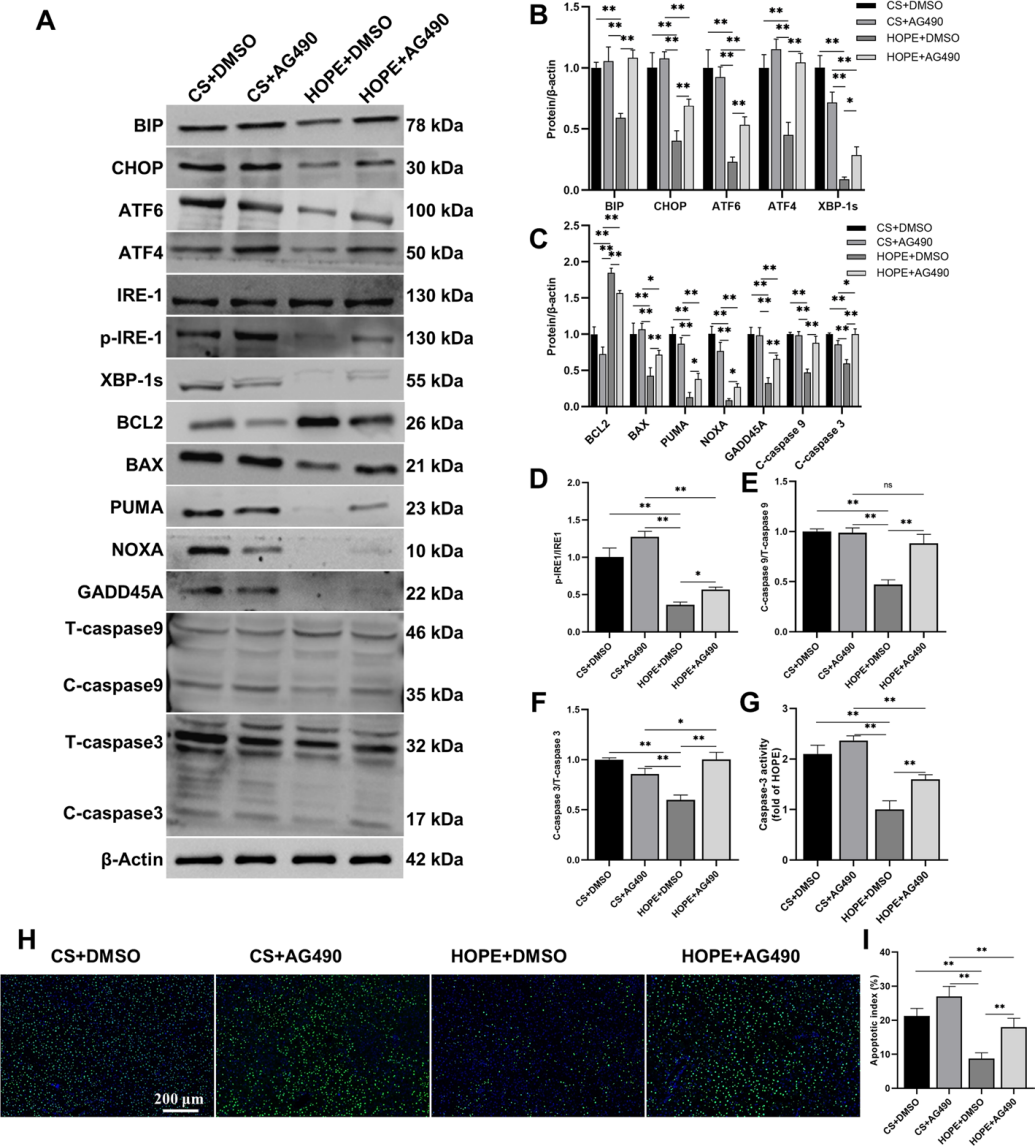
HOPE attenuates ER stress and apoptosis by activating JAK2/STAT3 pathway in a rat model of DCD. **A**–**F** Western blotting and statistical analyses of BIP, CHOP, ATF6, ATF4, IRE1, p-IRE1, XBP-1s, BCL2, BAX, PUMA, NOXA, GADD45A, caspase-9, caspase-3, and β-actin proteins (*n* = 3 for each group). p-IRE1 indicates phospho-IRE1, T-caspase indicates total caspase, C-caspase indicates cleaved caspase. **G** Caspase-3 activity detection (*n* = 6 for each group). **H**, **I** TUNEL staining and scoring of rat liver tissue (*n* = 6 for each group). Scale = 200 μm. ^*^p < 0.05 and ^**^p < 0.01

Next, we investigated whether the JAK2/STAT3 pathway regulates ER stress and apoptosis in a DCD rat model. Compared with the CS group, HOPE treatment significantly decreased the expression of ER stress marker proteins CHOP and BIP, and key signaling molecules ATF6, ATF4, p-IRE1, and XBP-1s in the three UPR pathways. The effect of HOPE treatment on the expression of these proteins was eliminated or weakened by AG490 (Fig. 5A–F). In addition, the effect of HOPE on the apoptosis-related proteins BCL2, BAX, PUMA, NOXA, GADD45A, Cleaved-caspase-9, and Cleaved-caspase-3 was reversed by AG490 (Fig. 5A–F). The change in caspase-3 activity was similar to its protein expression, showing a decrease under HOPE treatment that was subsequently reversed by AG490 (Fig. 5G). TUNEL staining also confirmed that AG490 reversed the antiapoptotic effects of HOPE (Fig. 5H, I). These results suggest that HOPE reduces ER stress and apoptosis by activating the JAK2/STAT3 pathway in livers of DCD rat model.

### JAK2/STAT3 pathway regulates the interaction between HAX1 and SERCA2b

**Fig. 6:**
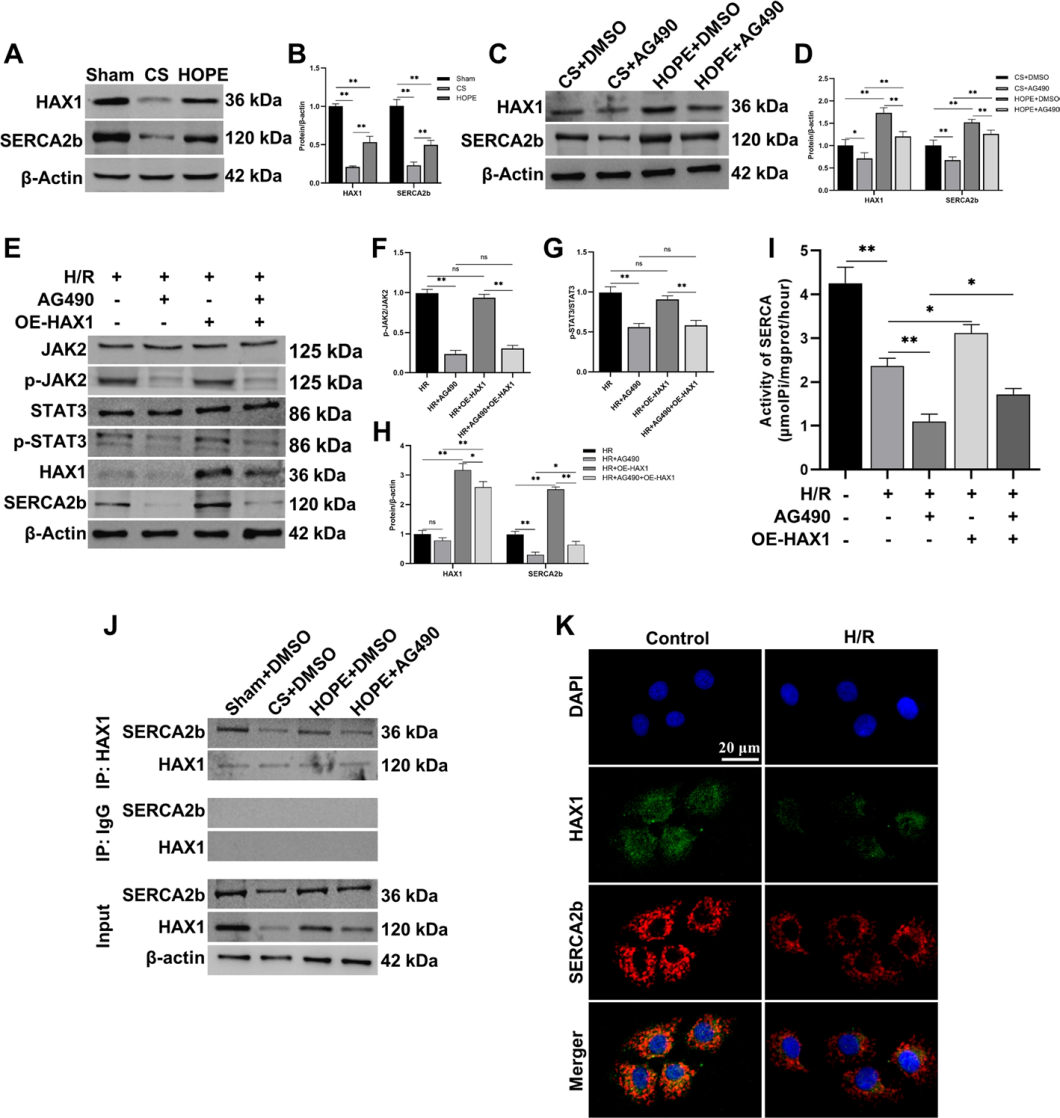
JAK2/STAT3 pathway regulates the interaction between HAX1 and SERCA2b. **A**, **B** Western blotting and statistical analyses of HAX1, SERCA2b, and β-actin proteins in rat liver tissues (*n* = 3 for each group). **C**, **D** Western blotting and statistical analyses of HAX1, SERCA2b, and β-actin proteins after AG490 pretreatment in rat liver tissues (*n* = 3 for each group). **E**–**H** Western blotting and statistical analyses of JAK2, p-JAK2, STAT3, p-STAT3, HAX1, SERCA2b, and β-actin proteins after AG490 and OE-HAX1 treatment in H/R cell model of BRL-3A (*n* = 3 for each group). p-JAK2 indicates phospho-JAK2, p-STAT3 indicates phospho-STAT3, OE-HAX1 indicates overexpressed HAX1. **I** SERCA activity analysis after AG490 and OE-HAX1 treatment in H/R cell model of BRL-3A (*n* = 6 for each group). **J** CO-IP analysis of the interaction between HAX1 and SERCA2b in rat liver tissues (*n* = 3 for each group). **K** Co-staining of HAX1 (green) with SERCA2b (red) in BRL-3A cell model (*n* = 6 for each group). Scale = 20 μm. ^*^p < 0.05 and ^**^p < 0.01

Hematopoietic-lineage substrate-1-associated protein X-1 (HAX1) is an antiapoptotic protein that mainly exists in the mitochondrial and ER membranes [45]. Several studies have demonstrated that HAX1 plays a key role in cell survival [46]. HAX1 can regulate ER Ca^2+^ homeostasis and the IRE1 pathway to reduce myocardial ER stress [47, 48]. In addition, HAX1 may inhibit apoptosis by blocking the activation of caspase-9 and caspase-3 [49]. Impaired SERCA activity can induce ER stress [22, 23]. Therefore, we investigated the effects of HOPE on the expression of HAX1 and SERCA2b. The results indicated that compared with the sham group, the expression levels of HAX1 and SERCA2b in the DCD liver significantly decreased after CS, and some of them reversed after HOPE treatment (Fig. 6A, B). Thus, HOPE promotes the expression of HAX1 and SERCA2b in DCD liver IRI.

To explore whether the JAK2/STAT3 pathway mediated these changes via HAX1 and SERCA2b expression and to investigate the role of these proteins in HOPE, we used AG490 as an inhibitor of the JAK2/STAT3 pathway. The results indicate that HAX1 and SERCA2b were downregulated after the addition of AG490 in both CS and HOPE groups. In addition, compared with CS, HOPE promoted the expression of HAX1 and SERCA2b with or without AG490 (Fig. 6C, D). Therefore, we concluded that the effects of HOPE on HAX1 and SERCA2b were mediated by the JAK2/STAT3 pathway. To further verify that the JAK2/STAT3 pathway regulates HAX1 and SERCA2b expression, we constructed an H/R cell model of BRL-3A. The results showed that after inhibition of the JAK2/STAT3 pathway by AG490, HAX1 and SERCA2b was downregulated, but this downregulation was not statistically significant in the case of HAX1 (Fig. 6E–H). We then constructed an overexpression plasmid for HAX1 and transfected BRL-3A cells in vitro. Interestingly, in the H/R cell model, the expression of SERCA2b increased immediately after HAX1 overexpression. SERCA2b expression also increased after overexpression of HAX1 under the action of AG490 (Fig. 6E–H). In addition, AG490 significantly inhibited the activity of SERCA, while this inhibitory effect was attenuated by HAX1 overexpression (Fig. 6I). This further indicates that the JAK2/STAT3 pathway affects the expression of HAX1 and SERCA2b, and that HAX1 can regulate the expression and activity of SERCA2b.

Next, we investigated whether HAX1 and SERCA2b interacted. First, CO-IP analysis was performed in a rat model of DCD. Compared with that in the sham group, the inhibition of the interaction between HAX1 and SERCA2b by CS was significantly alleviated by HOPE. However, the action of AG490 nullified the action of HOPE (Fig. 6J). In addition, H/R treatment of BRL-3A significantly weakened the colocalization of HAX1 and SERCA2b (Fig. 6K). The interaction between HAX1 and SERCA2b was confirmed in vivo and in vitro and was regulated by JAK2/STAT3.

### JAK2/STAT3 pathway regulates ER stress and apoptosis through HAX1

**Fig. 7:**
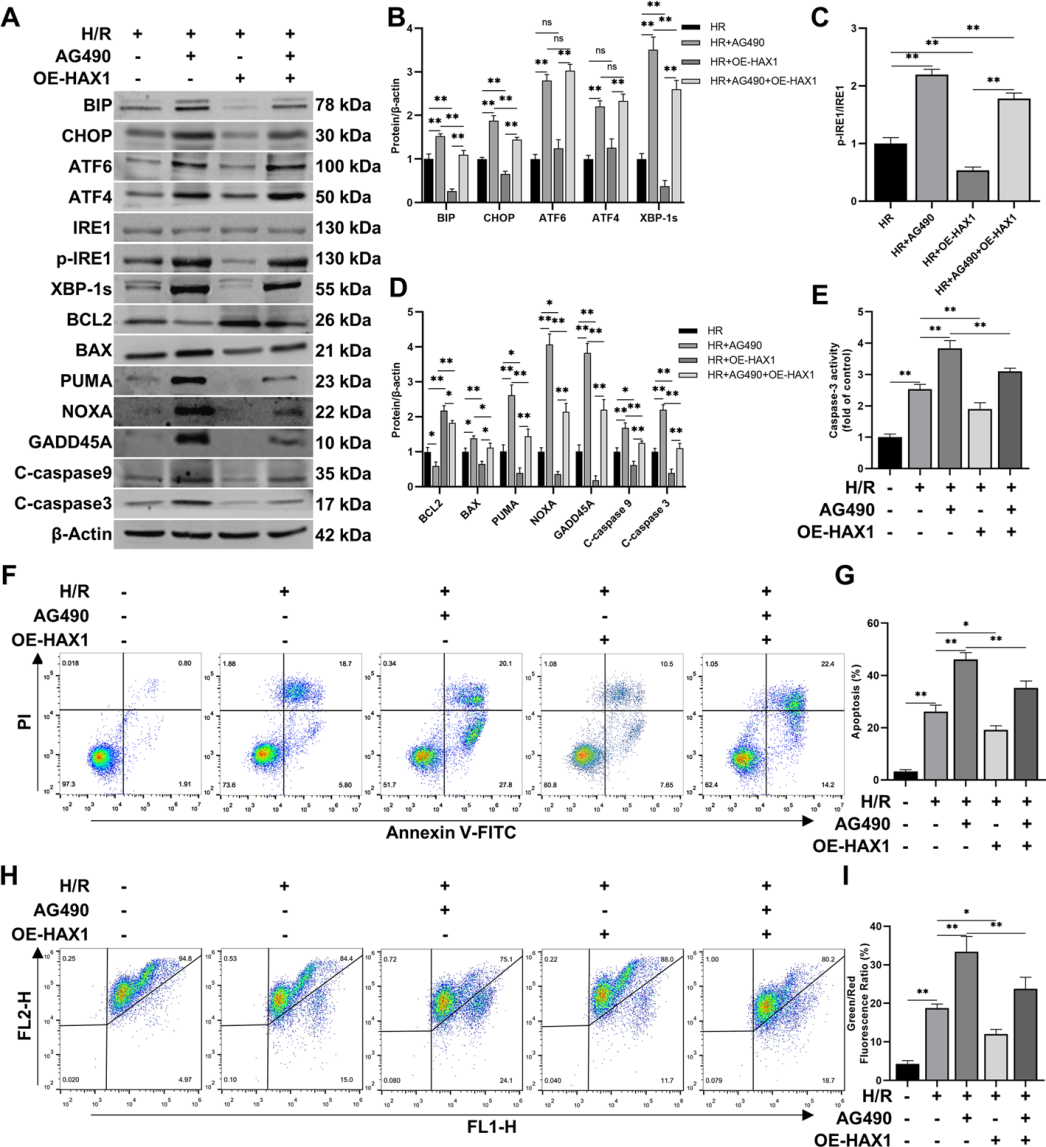
JAK2/STAT3 pathway regulates ER stress and apoptosis through HAX1. **A**–**D** Western blotting and statistical analyses of BIP, CHOP, ATF6, ATF4, IRE1, p-IRE1, XBP-1s, BCL2, BAX, PUMA, NOXA, GADD45A, caspase-9, caspase-3, and β-actin proteins after AG490 and OE-HAX1 treatment in H/R cell model of BRL-3A (*n* = 3 for each group). p-IRE1 indicates phospho-IRE1, C-caspase indicates cleaved caspase, OE-HAX1 indicates overexpressed HAX1. **E** Caspase-3 activity detection (*n* = 6 for each group). **F**, **G** Flow cytometric analysis used for the detection of apoptosis after AG490 and OE-HAX1 treatment in H/R cell model of BRL-3A (*n* = 3 for each group). **H**, **I** Flow cytometric analysis used for the detection of mitochondrial membrane potential in H/R cell model of BRL-3A (*n* = 3 for each group). ^*^p < 0.05 and ^**^p < 0.01

To verify whether the JAK2/STAT3 pathway regulates HAX1 to reduce ER stress and apoptosis, we evaluated the expression of related proteins in BRL-3A cells. The results showed that after inhibiting the JAK2/STAT3 pathway by AG490, the expression of ER stress and UPR-related proteins BIP, CHOP, ATF6, ATF4, p-IRE1, and XBP-1s increased, while the expression of apoptosis-related protein BCL2 decreased, and BAX, PUMA, NOXA, GADD45A, C-caspase-9, and C-caspase-3 increased. However, after HAX1 overexpression, CHOP and BIP were downregulated. Among the three UPR pathways, ATF6 and ATF4 (key molecules of the PERK pathway) showed no significant change. Only the phosphorylation level of IRE1 decreased, and the expression of XBP-1s was inhibited (Fig. 7A–D). This indicated that HAX1 regulates ER stress primarily through the IRE1 pathway. After overexpressing HAX1, the expression of BCL2 increased, while that of BAX, PUMA, NOXA, GADD45A, C-caspase-9, and C-caspase-3 decreased (Fig. 7A–D). Moreover, the effects of HAX1 overexpression on ER stress and apoptosis-related proteins were reversed by AG490 (Fig. 7A–D). The change in caspase-3 activity was similar to that of its protein expression (Fig. 7E). Flow cytometry analysis of the HR cell model showed that AG490 increased the apoptosis rate of cells, while HAX1 overexpression reduced the apoptosis rate (Fig. 7F, G). Mitochondria serve as the key regulators of cell apoptosis, playing a crucial role in initiating cell death [50]. In this regard, we examined the mitochondrial membrane potential and observed that AG490 reduced it. However, when HAX1 was overexpressed, it can mitigate the AG490-induced decline in mitochondrial membrane potential (Fig. 7H, I). These findings suggest that ER stress induces cell apoptosis via mitochondrial processes, which are dependent on HAX1. These results suggest that the JAK2/STAT3 pathway can reduce ER stress and apoptosis by regulating the HAX1/IRE1 pathway.

## Discussion

IRI in DCD liver transplantation is an important factor that affects donor liver quality and recipient survival rate [32]. MP is currently the focus of liver transplantation research, and HOPE is widely used for marginal liver transplantation (including DCD and steatosis liver) [16]. Acellular perfusion containing dissolved oxygen appears to be sufficient to maintain cell function at low temperatures due to the low demand for high-energy substances and the lack of oxygen carriers [15, 51]. In addition, the release of AST/ALT in the perfusate of high-risk DCD liver during HOPE was lower than that during normothermic perfusion [52, 53]. In this study, we also found that HOPE improved the function of the DCD liver and reduced ER stress and apoptosis by regulating the JAK2/STAT3/HAX1 signaling pathway. Therefore, HOPE can not only prevent IRI and improve liver function but can also be used as a tool to predict early graft function of the receptor.

The ER is critical to the proper physiological functioning of eukaryotic cells. As the main organelle for protein synthesis, storage, processing, modification, folding, assembly, and transport, the ER largely maintains the proper physiological function of eukaryotic cells [21]. The liver is the largest metabolic organ in the human body, enzyme-rich, with metabolic and secretory activities, and crucial for protein synthesis. Hepatocytes have a well-developed ER system, aiding the liver in carrying out its physiological function [54]. ER stress has been linked to several phases of liver injury, and hepatocyte death mediated by ER stress may be the key to liver injury [55]. IRI in the liver stimulates the ER, disrupts Ca^2+^, and destroys ER homeostasis [54]. The accumulation of unfolded proteins leads to the formation of the UPR. Activation of the UPR signaling pathway directly leads to apoptosis [56]. PERK, ATF6, and IRE1 pathways induce CHOP expression, inhibiting BCL2 and promoting apoptotic protein expression [29]. In our study, the ER stress marker proteins BIP and CHOP, as well as key molecules of the three UPR signaling pathways (ATF6, ATF4, p-IRE1, and XBP-1s), were upregulated during the IRI process in the DCD liver, and HOPE significantly inhibited the expression of these proteins, inhibiting apoptosis. This suggests that HOPE can alleviate ER stress and apoptosis by inhibiting the three UPR pathways (PERK, ATF6, and IRE1). Increasing evidence has shown that the regulation of ER stress has great potential for treating liver injury. Our study is the first to report the use of HOPE to regulate ER stress during IRI in the DCD liver, further demonstrating that HOPE has great potential in reducing liver injury caused by DCD.

Mitochondria serve as the central regulators of cell apoptosis[50]. Excessive ER stress can induce cell apoptosis through various pathways, either dependent or independent of mitochondria. These pathways involve processes such as mitochondrial membrane pore opening, mitochondrial depolarization, generation of reactive oxygen species (ROS), and activation of caspase [57]. The interplay between the ER and mitochondria plays a crucial role in cellular phenomena associated with cell death, including dynamic changes in intercellular structure and function [58–60]. Several signaling molecules, such as BCL2 family proteins, kinases, proteases, and transcription factors, are implicated in mediating cell apoptosis triggered by ER stress [47]. In our study, we observed upregulation of BCL2 family-related proteins (PUMA, NOXA), the DNA damage sensor GADD45A, as well as caspase-3 and caspase-9 in the CS group. However, treatment with HOPE significantly suppressed the expression of these proteins, suggesting that HOPE may mitigate ER stress-induced cell apoptosis by modulating mitochondrial events.

The JAK2/STAT3 pathway plays an essential role in antiinflammation, antioxidant stress, and antiapoptosis [61, 62]. In this study, HOPE induced the phosphorylation of JAK2 and STAT3 in the livers of DCD rat models. Treatment with AG490, an inhibitor of the JAK2/STAT3 pathway, reversed the positive effect of HOPE on DCD livers. Interestingly, after inhibition of the JAK2/STAT3 pathway, the effects of HOPE on BIP, CHOP, ATF6, ATF4, p-IRE1, XBP-1s, BCL2, BAX, PUMA, NOXA, GADD45A, caspase-9, and caspase-3 were also inhibited. This further proves that HOPE has a protective effect on rat DCD livers by activating the JAK2/STAT3 pathway to inhibit the UPR pathways (PERK, ATF6, and IRE1 pathways) and apoptosis.

HAX1 is an antiapoptotic protein widely expressed in human tissues, mainly distributed in the mitochondria, ER, and cytoplasm near the nuclear membrane, playing various roles [45]. In mitochondria, HAX1 regulates cyclophilin-D levels through a heat shock protein-90 (HSP90)-dependent mechanism, which protects against the activation of the mitochondrial permeability transition pore (mPTP) and subsequent cell death [46]. HAX1 is also involved in ER stress, mainly by regulating the IRE1 pathway, including its downstream effect factors CHOP [47, 48]. In the immune system, the functional CLPB/HAX1/PRKD2/HSP27-dependent axis is involved in maintaining mitochondrial protein balance and neutrophil differentiation [63]. In cardiomyocytes, HAX1 interacts with PLN in the ER, regulates Ca^2+^ homeostasis in the ER, and inhibits ER stress [48]. SERCA is a Ca^2+^ channel in the ER, and SERCA2b is the predominant subtype in hepatocytes [24]. Inhibition of the expression and activity of SERCA2b leads to decreased Ca^2+^ storage in the ER and cytoplasmic Ca^2+^ overload, resulting in ER stress [64]. In this study, compared with that in the CS group, HAX1 and SERCA2b was upregulated, and the activity of SERCA was enhanced after HOPE treatment. This positive effect of HOPE was significantly reversed by AG490. We studied the interactions between HAX1 and SERCA2b. The results of CO-IP and co-localization experiments showed an interaction between them. Compared with that under CS treatment, the interaction was significantly enhanced under HOPE treatment but was inhibited by AG490. This indicates that HOPE regulates HAX1/SERCA2b interaction by activating the JAK2/STAT3 pathway to maintain ER calcium homeostasis. When we overexpressed HAX1 in the H/R model of BRL-3A cells, the effects of AG490 on BIP, CHOP, p-IRE1, XBP-1s, BCL2, BAX, PUMA, NOXA, GADD45A, caspase-9, and caspase-3 were partially eliminated, whereas ATF6 and ATF4 (key molecules in the PERK pathway) showed no significant changes. This indicates that the regulation of the UPR pathway by HAX1 occurs through the IRE1 pathway rather than the PERK or ATF6 pathways. Therefore, it can be concluded that HOPE upregulates HAX1 by activating the JAK2/STAT3 signaling pathway. On the one hand, HAX1 stabilizes ER calcium homeostasis by regulating the interaction of HAX1/SERCA2b. On the other hand, upregulated HAX1 reduces ER stress and apoptosis by inhibiting the IRE1 pathway, protecting the liver from IRI in rat DCD liver. Unfortunately, as HOPE is not yet an established clinical practice, our conclusions based on animal experiments need to be further validated before its clinical applications.

## Conclusions

**Fig. 8:**
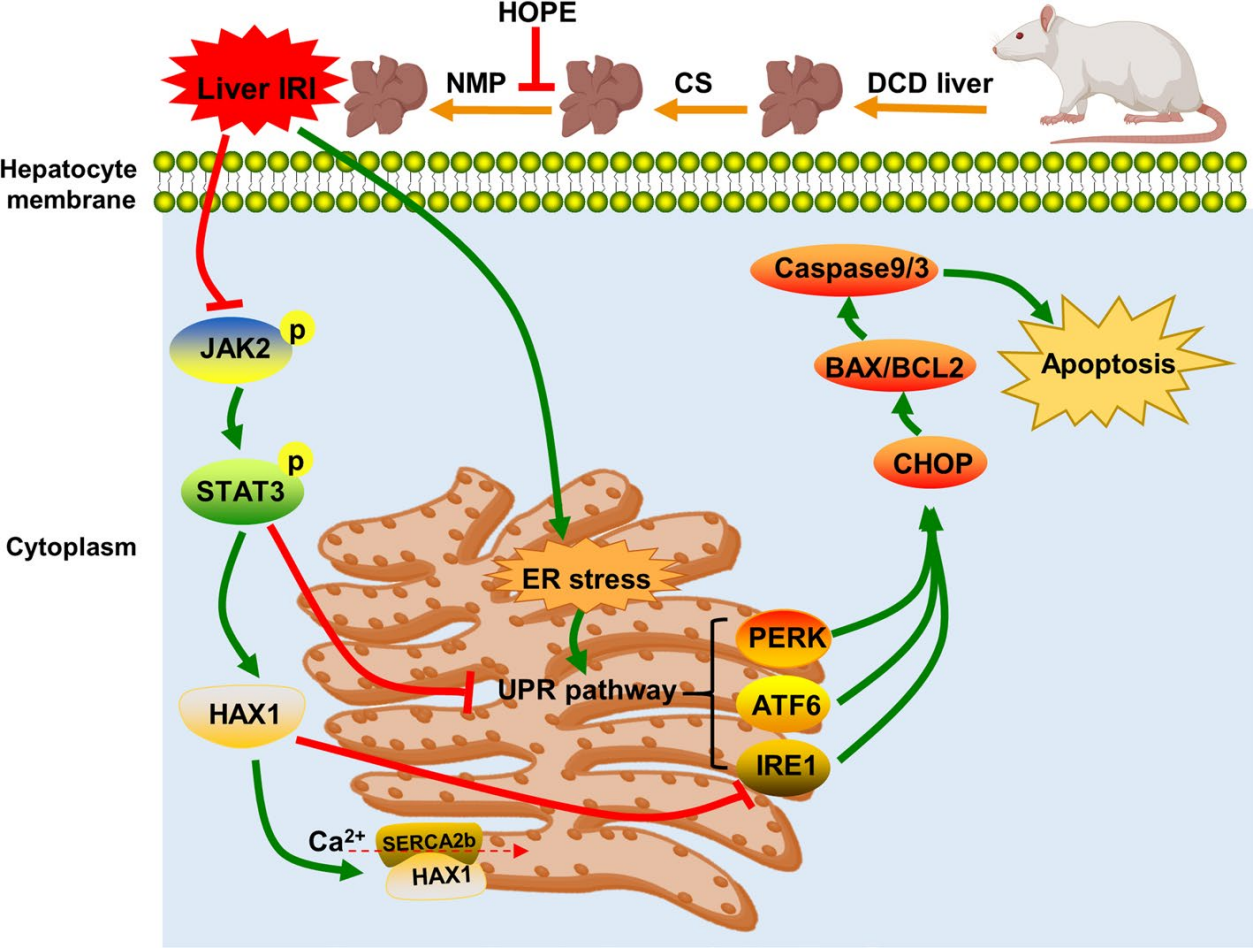
Schematic showing proposed mechanism. HOPE inhibits the three branches of UPR (PERK, ATF6, and IRE1 pathways) by activating JAK2/STAT3 signal pathway, thus alleviating ER stress and apoptosis. In addition, activated JAK2/STAT3 signal pathway can maintain ER calcium homeostasis by upregulating HAX1 and promoting the interaction of HAX1/SERCA2b. Upregulated HAX1 also regulates ER stress and apoptosis by inhibiting the IRE1 pathway

Our research shows that HOPE inhibits the UPR pathways (PERK, ATF6, and IRE1 pathways) by activating JAK2/STAT3, thereby exerting a protective effect on DCD liver IRI in rats. Activated JAK2/STAT3 maintains calcium homeostasis in the ER by upregulating HAX1 and promoting the interaction between HAX1/SERCA2b. Upregulated HAX1 also inhibits ER stress and apoptosis by regulating the IRE1 pathway (Fig. 8). Therefore, targeting the JAK2/STAT3/HAX1 signaling pathway using HOPE can be an effective strategy for the treatment of IRI during DCD liver transplantation.

## Supplementary Information

**Additional file 1: Table S1.** Primer sequences of the target genes.

**Additional file 2: Fig. S1.** mRNA expression levels of BIP and CHOP.

## Data Availability

The data presented in this study can be obtained upon reasonable request from the corresponding author.

## Abbreviations

IRI: Ischemia–reperfusion injury
DCD: Donation after cardiac death
ECD: Extended criteria donor
EAD: Early allograft dysfunction
PNF: Primary nonfunction
ER: Endoplasmic reticulum
JAK2: Janus kinase 2
STAT3: Signal transducer and activator of transcription 3
HAX1: Hematopoietic-lineage substrate-1-associated protein X-1
CS: Cold storage
HOPE: Hypothermic oxygenated perfusion
NMP: Normothermic perfusion
UPR: Unfolded protein response
PERK: Protein kinase RNA-like ER kinase
ATF6: Activating transcription factor 6
IRE1: Inositol-requiring enzyme 1
XBP-1s: X-box binding protein l splicing
SERCA: Sarco/endoplasmic reticulum calcium ATPase
BIP: Binding protein
CHOP: C/EBP homologous protein
BCL2: B-cell lymphoma 2
PUMA: P53-upregulated modulator of apoptosis
AST: Alanine aminotransferase
ALT: Aspartate aminotransferase
H&E: Hematoxylin and eosin
IHC: Immunohistochemistry
IF: Immunofluorescence
CO-IP: Co-immunoprecipitation
