# Supplementary material for: Hypothermic oxygenated perfusion attenuates DCD liver ischemia–reperfusion injury by activating the JAK2/STAT3/HAX1 pathway to regulate endoplasmic reticulum stress
Source: Cell Mol Biol Lett. 2023 Jul 12;28:55. doi: 10.1186/s11658-023-00466-5 (PMC10337067; doi:10.1186/s11658-023-00466-5)
Supplement: Supplementary file 1 — Additional file 1: Table S1. Primer sequences of the target genes. [file 11658_2023_466_MOESM1_ESM.docx]

Additional file 1: Table S1. Primer sequences of the target genes

| **Gene name** | **Primer name** | **Primer sequences (5′-3′)** |
| --- | --- | --- |
| BIP | Forward  Reverse | GCCTTTTGCGGGTTTGAGAG  TAGGTGGTCCCCAAGTCGAT |
| CHOP | Forward  Reverse | GAACCTGAGGAGAGAGTGTTCC  CTCATACCAGGCTTCCAGCTC |
| IL-1β | Forward  Reverse | AGCTTCAGGAAGGCAGTGTC  TCAGACAGCACGAGGCATTT |
| IL-2 | Forward  Reverse | TGTCCTCCTTGTCAACAGCG  AATTCTGTGGCCTGCTTGGG |
| IL-4 | Forward  Reverse | GTACCGGGAACGGTATCCAC  GTGAGTTCAGACCGCTGACA |
| IL-6 | Forward  Reverse | ACAAGTCCGGAGAGGAGACT  ACAGTGCATCATCGCTGTTC |
| IL-10 | Forward  Reverse | TCCCTGGGAGAGAAGCTGAA  GTAGATGCCGGGTGGTTCAA |
| TNF-α | Forward  Reverse | AAGCTGTCTTCAGGCCAACA  CCCGTAGGGCGATTACAGTC |
| IFN-γ | Forward  Reverse | GGCAAAAGGACGGTAACACG  TCTGTGGGTTGTTCACCTCG |
| HMGB1 | Forward  Reverse | AACAACACTTGGTGCTGGGC  TCCTCCCAGGGCTTAAGAGAA |
| β-Actin | Forward  Reverse | ACCCGCGAGTACAACCTTCT  GCCGTGTTCAATGGGGTACT |
