# Supplementary figures and images for: Hypothermic oxygenated perfusion attenuates DCD liver ischemia–reperfusion injury by activating the JAK2/STAT3/HAX1 pathway to regulate endoplasmic reticulum stress
Source: Cell Mol Biol Lett. 2023 Jul 12;28:55. doi: 10.1186/s11658-023-00466-5 (PMC10337067; doi:10.1186/s11658-023-00466-5)

**Additional file 2**


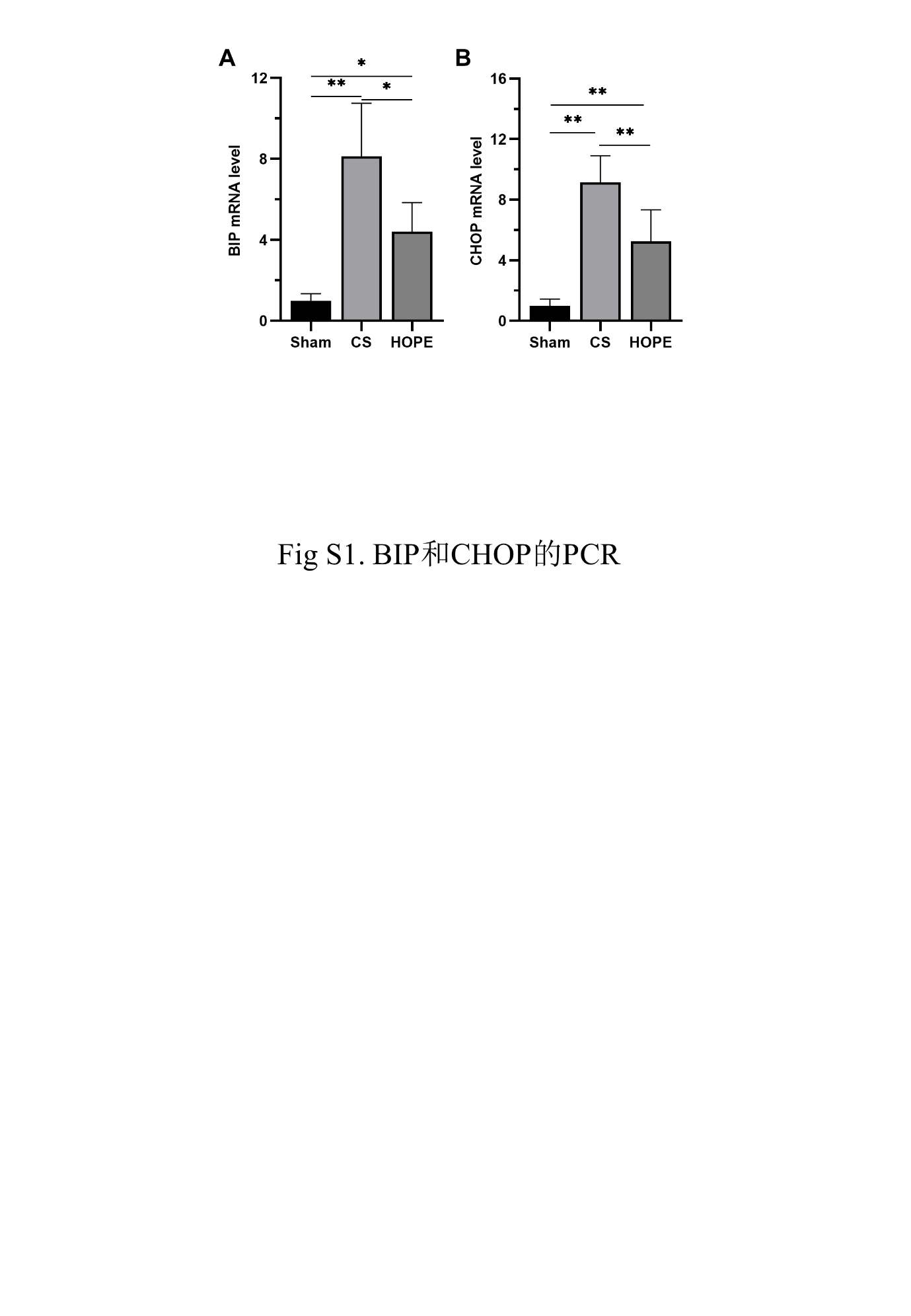


**Fig. S1** mRNA expression level of BIP (**A**) and CHOP (**B**). **p* < 0.05 and ** *p* < 0.01.

Supplement: Supplementary file 2 — Additional file 2: Fig. S1. mRNA expression levels of BIP and CHOP. [file 11658_2023_466_MOESM2_ESM.docx]
